# Penta­aqua­(1*H*-benzimidazole-5,6-dicarboxyl­ato-κ*N* ^3^)nickel(II) penta­hydrate

**DOI:** 10.1107/S1600536809018704

**Published:** 2009-05-23

**Authors:** Wen-Dong Song, Hao Wang, Pei-Wen Qin, Shi-Jie Li, Shi-Wei Hu

**Affiliations:** a College of Science, Guang Dong Ocean University, Zhanjiang 524088, People’s Republic of China

## Abstract

In the title mononuclear complex, [Ni(C_9_H_4_N_2_O_4_)(H_2_O)_5_]·5H_2_O, the Ni^II^ atom is six-coordinated by one N atom from a 1*H*-benzimidazole-5,6-dicarboxyl­ate ligand and by five O atoms from five water mol­ecules and displays a distorted octa­hedral geometry. Inter­molecular O—H⋯O hydrogen-bonding inter­actions among the coordinated water mol­ecules, solvent water mol­ecules and carboxyl O atoms of the organic ligand and additional N—H⋯O hydrogen bonding lead to the formation of a three-dimensional supra­molecular network.

## Related literature

For background information on 1*H*-benzimidazole-5,6-dicarboxyl­ate complexes, see: Lo *et al.* (2007 ▶); Yao *et al.* (2008 ▶).
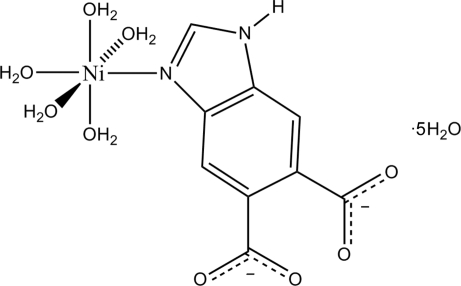


## Experimental

### 

#### Crystal data

[Ni(C_9_H_4_N_2_O_4_)(H_2_O)_5_]·5H_2_O
*M* *_r_* = 443.01
Triclinic, 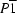

*a* = 6.8436 (14) Å
*b* = 11.434 (2) Å
*c* = 12.344 (3) Å
α = 78.29 (3)°
β = 78.65 (3)°
γ = 74.92 (3)°
*V* = 902.6 (3) Å^3^
*Z* = 2
Mo *K*α radiation
μ = 1.15 mm^−1^
*T* = 293 K
0.31 × 0.25 × 0.21 mm

#### Data collection

Rigaku Mercury CCD diffractometer
Absorption correction: multi-scan (*REQAB*; Jacobson, 1998 ▶) *T*_min_ = 0.725, *T*_max_ = 0.793
7176 measured reflections
3228 independent reflections
2851 reflections with *I* > 2σ(*I*)
*R*_int_ = 0.048

#### Refinement

*R*[*F* ^2^ > 2σ(*F* ^2^)] = 0.056
*wR*(*F* ^2^) = 0.167
*S* = 1.14
3228 reflections
235 parameters
30 restraints
H-atom parameters constrained
Δρ_max_ = 1.53 e Å^−3^
Δρ_min_ = −0.60 e Å^−3^

### 

Data collection: *RAPID-AUTO* (Rigaku, 1998 ▶); cell refinement: *RAPID-AUTO*; data reduction: *CrystalStructure* (Rigaku/MSC, 2002 ▶); program(s) used to solve structure: *SHELXS97* (Sheldrick, 2008 ▶); program(s) used to refine structure: *SHELXL97* (Sheldrick, 2008 ▶); molecular graphics: *ORTEPII* (Johnson, 1976 ▶); software used to prepare material for publication: *SHELXL97*.

## Supplementary Material

Crystal structure: contains datablocks I, global. DOI: 10.1107/S1600536809018704/zl2208sup1.cif

Structure factors: contains datablocks I. DOI: 10.1107/S1600536809018704/zl2208Isup2.hkl

Additional supplementary materials:  crystallographic information; 3D view; checkCIF report

## Figures and Tables

**Table 1:** Hydrogen-bond geometry (Å, °)

*D*—H⋯*A*	*D*—H	H⋯*A*	*D*⋯*A*	*D*—H⋯*A*
O10*W*—H20*W*⋯O1*W*	0.84	2.00	2.836 (4)	176
O10*W*—H19*W*⋯O8*W*^i^	0.84	1.88	2.703 (5)	166
O9*W*—H17*W*⋯O3^ii^	0.84	1.90	2.733 (5)	172
O9*W*—H18*W*⋯O10*W*^iii^	0.84	1.91	2.720 (5)	163
O8*W*—H15*W*⋯O1^iv^	0.84	1.95	2.765 (5)	163
O8*W*—H16*W*⋯O2	0.84	1.96	2.775 (5)	162
O7*W*—H13*W*⋯O8*W*^v^	0.84	1.93	2.754 (5)	165
O7*W*—H14*W*⋯O4^v^	0.84	1.91	2.734 (5)	169
O6*W*—H12*W*⋯O2*W*^vi^	0.84	2.06	2.857 (4)	159
O6*W*—H11*W*⋯O4^vii^	0.84	1.97	2.808 (4)	174
O5*W*—H10*W*⋯O4^viii^	0.84	1.96	2.800 (4)	176
O5*W*—H9*W*⋯O9*W*^iii^	0.84	1.98	2.817 (4)	173
O4*W*—H8*W*⋯O9*W*^v^	0.84	1.90	2.736 (5)	173
O4*W*—H7*W*⋯O3^ix^	0.84	1.94	2.709 (4)	151
O3*W*—H6*W*⋯O6*W*^viii^	0.84	1.93	2.761 (4)	172
O3*W*—H5*W*⋯O7*W*^x^	0.84	1.93	2.729 (5)	159
O2*W*—H4*W*⋯O1^v^	0.84	1.80	2.620 (4)	164
O2*W*—H3*W*⋯O10*W*^iv^	0.84	1.90	2.734 (5)	175
O1*W*—H1*W*⋯O6*W*^v^	0.84	1.96	2.783 (5)	168
O1*W*—H2*W*⋯O2^v^	0.84	1.79	2.612 (4)	166
N1—H1⋯O7*W*^xi^	0.86	1.97	2.803 (5)	162

Symmetry codes: (i) 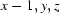; (ii) 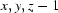; (iii) 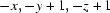; (iv) 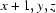; (v) 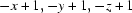; (vi) 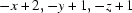; (vii) 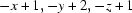; (viii) 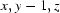; (ix) 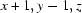; (x) 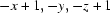; (xi) 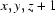.

**Fig. 1.**
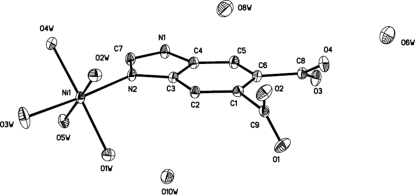
The structure of the title compound, showing the atomic numbering scheme. Non-H atoms are shown with 30% probability displacement ellipsoids.

**Fig. 2.**
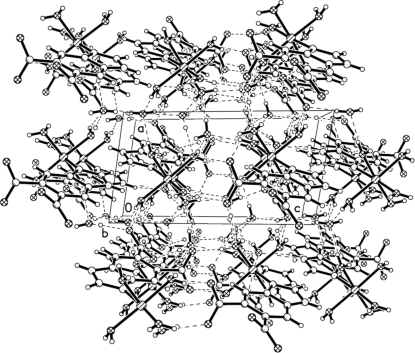
A packing view of the title compound. The intermolecular hydrogen bonds are shown as dashed lines.

## supplementary crystallographic information

### Comment

In the structural investigation of 1*H*-benzimidazole-5,6-dicarboxylate complexes, it has been found that the 1*H*-benzimidazole-5,6-dicarboxylic acid can function as a multidentate ligand (Lo *et al.*, 2007; Yao *et al.*, 2008), with versatile binding and coordination modes. In this paper, we report the crystal structure of the title compound, a new Ni complex obtained by the reaction of 1*H*-benzimidazole-5,6-dicarboxylic acid with nickel chloride in an alkaline aqueous solution.

As illustrated in Fig. 1, the Ni^II^ atom exhibits a slightly distorted octahedral coordination sphere, defined by one N atom from the 1*H*-benzimidazole-5,6-dicarboxylate ligand and five coordinated water molecules. The five non-bonded solvent water molecules are located in cavities of the three-dimensional framework, allowing them to participate in various O—H···O hydrogen bonds (Table 1) with the coordinated water molecules, non-coordinated water molecules and carboxylate O atoms of the organic ligand. The hydrogen bonds are in the normal range (Table 1, Fig. 2).

### Experimental

A mixture of nickel chloride (1 mmol), 1*H*-benzimidazole-5,6-dicarboxylic acid (1 mmol), NaOH (1.5 mmol) and H_2_O (12 ml) was placed in a 23 ml Teflon reactor, which was heated to 433 K for three days and then cooled to room temperature at a rate of 10 K h^-1^. The crystals obtained were washed with water and dryed in air.

### Refinement

Carbon and nitrogen bound H atoms were placed at calculated positions and were treated as riding on the parent C or N atoms with C—H = 0.93 Å, N—H = 0.86 Å, and with *U*_iso_(H) = 1.2 *U*_eq_(C, N). The water H atoms were located in a difference map, and were refined with a distance restraint of O—H = 0.84 Å; their *U*_iso_ values were refined.

### Crystal data

**Table:**

[Ni(C_9_H_4_N_2_O_4_)(H_2_O)_5_]·5H_2_O	*Z* = 2
*M**_r_* = 443.01	*F*(000) = 464
Triclinic, *P*1	*D*_x_ = 1.630 Mg m^−^^3^
Hall symbol: -P 1	Mo *K*α radiation, λ = 0.71073 Å
*a* = 6.8436 (14) Å	Cell parameters from 3600 reflections
*b* = 11.434 (2) Å	θ = 1.4–28°
*c* = 12.344 (3) Å	µ = 1.15 mm^−^^1^
α = 78.29 (3)°	*T* = 293 K
β = 78.65 (3)°	Block, blue
γ = 74.92 (3)°	0.31 × 0.25 × 0.21 mm
*V* = 902.6 (3) Å^3^	

### Data collection

**Table:**

Rigaku Mercury CCD diffractometer	3228 independent reflections
Radiation source: fine-focus sealed tube	2851 reflections with *I* > 2σ(*I*)
graphite	*R*_int_ = 0.048
ω scans	θ_max_ = 25.2°, θ_min_ = 3.1°
Absorption correction: multi-scan (REQAB; Jacobson, 1998)	*h* = −8→8
*T*_min_ = 0.725, *T*_max_ = 0.793	*k* = −13→13
7176 measured reflections	*l* = −14→13

### Refinement

**Table:**

Refinement on *F*^2^	Primary atom site location: structure-invariant direct methods
Least-squares matrix: full	Secondary atom site location: difference Fourier map
*R*[*F*^2^ > 2σ(*F*^2^)] = 0.056	Hydrogen site location: inferred from neighbouring sites
*wR*(*F*^2^) = 0.167	H-atom parameters constrained
*S* = 1.14	*w* = 1/[σ^2^(*F*_o_^2^) + (0.0905*P*)^2^ + 1.2897*P*] where *P* = (*F*_o_^2^ + 2*F*_c_^2^)/3
3228 reflections	(Δ/σ)_max_ = 0.001
235 parameters	Δρ_max_ = 1.53 e Å^−^^3^
30 restraints	Δρ_min_ = −0.60 e Å^−^^3^

### Special details

**Table:**

Geometry. All e.s.d.'s (except the e.s.d. in the dihedral angle between two l.s. planes) are estimated using the full covariance matrix. The cell e.s.d.'s are taken into account individually in the estimation of e.s.d.'s in distances, angles and torsion angles; correlations between e.s.d.'s in cell parameters are only used when they are defined by crystal symmetry. An approximate (isotropic) treatment of cell e.s.d.'s is used for estimating e.s.d.'s involving l.s. planes.
Refinement. Refinement of *F*^2^ against ALL reflections. The weighted *R*-factor *wR* and goodness of fit *S* are based on *F*^2^, conventional *R*-factors *R* are based on *F*, with *F* set to zero for negative *F*^2^. The threshold expression of *F*^2^ > σ(*F*^2^) is used only for calculating *R*-factors(gt) *etc*. and is not relevant to the choice of reflections for refinement. *R*-factors based on *F*^2^ are statistically about twice as large as those based on *F*, and *R*- factors based on ALL data will be even larger.

### Fractional atomic coordinates and isotropic or equivalent isotropic displacement parameters (Å^2^)

**Table:**

	*x*	*y*	*z*	*U*_iso_*/*U*_eq_	
C1	0.3714 (6)	0.5621 (4)	0.7112 (3)	0.0205 (8)	
N1	0.3498 (6)	0.3063 (3)	0.9967 (3)	0.0272 (8)	
H1	0.3074	0.3078	1.0669	0.033*	
Ni1	0.59930 (7)	0.09723 (4)	0.74101 (4)	0.0200 (2)	
O1	0.2171 (5)	0.6926 (3)	0.5614 (3)	0.0379 (8)	
C2	0.4484 (6)	0.4375 (3)	0.7118 (3)	0.0210 (8)	
H2	0.5106	0.4068	0.6460	0.025*	
N2	0.4903 (5)	0.2318 (3)	0.8396 (3)	0.0224 (7)	
O2	0.5536 (5)	0.6543 (3)	0.5459 (2)	0.0346 (8)	
C3	0.4301 (6)	0.3595 (3)	0.8136 (3)	0.0209 (8)	
O3	0.0491 (5)	0.7830 (3)	0.8792 (3)	0.0346 (8)	
C4	0.3381 (6)	0.4072 (4)	0.9121 (3)	0.0230 (8)	
O4	0.3074 (5)	0.8139 (3)	0.7441 (3)	0.0328 (7)	
C5	0.2592 (6)	0.5316 (4)	0.9124 (3)	0.0253 (9)	
H5	0.1973	0.5617	0.9785	0.030*	
C6	0.2755 (6)	0.6096 (4)	0.8113 (3)	0.0222 (8)	
C7	0.4385 (7)	0.2068 (4)	0.9496 (3)	0.0258 (9)	
H7	0.4618	0.1277	0.9898	0.031*	
C8	0.2025 (6)	0.7459 (4)	0.8107 (3)	0.0243 (9)	
C9	0.3812 (6)	0.6449 (3)	0.5979 (3)	0.0217 (8)	
O1W	0.3920 (4)	0.1803 (3)	0.6302 (2)	0.0284 (7)	
H2W	0.4303	0.2272	0.5727	0.043*	
H1W	0.3513	0.1237	0.6138	0.043*	
O2W	0.8180 (4)	0.1821 (3)	0.6393 (2)	0.0274 (6)	
H3W	0.8697	0.2273	0.6656	0.041*	
H4W	0.7925	0.2129	0.5744	0.041*	
O3W	0.7170 (6)	−0.0427 (3)	0.6518 (3)	0.0463 (10)	
H5W	0.7472	−0.1164	0.6823	0.070*	
H6W	0.7539	−0.0344	0.5822	0.070*	
O4W	0.7928 (5)	0.0082 (3)	0.8549 (3)	0.0313 (7)	
H7W	0.8647	−0.0596	0.8391	0.047*	
H8W	0.8618	0.0475	0.8765	0.047*	
O5W	0.3802 (5)	0.0021 (3)	0.8336 (2)	0.0285 (7)	
H9W	0.2734	0.0489	0.8607	0.043*	
H10W	0.3518	−0.0525	0.8072	0.043*	
O6W	0.8012 (5)	0.9831 (3)	0.4213 (2)	0.0340 (7)	
H11W	0.7615	1.0420	0.3717	0.051*	
H12W	0.9257	0.9499	0.4049	0.051*	
O7W	0.2893 (5)	0.2719 (3)	0.2313 (3)	0.0372 (8)	
H14W	0.4128	0.2554	0.2398	0.056*	
H13W	0.2201	0.3317	0.2624	0.056*	
O8W	0.9187 (5)	0.5590 (3)	0.6314 (3)	0.0431 (8)	
H16W	0.8200	0.5777	0.5952	0.065*	
H15W	1.0137	0.5932	0.5982	0.065*	
O9W	−0.0044 (5)	0.8476 (3)	0.0863 (3)	0.0378 (8)	
H18W	0.0184	0.7895	0.1395	0.057*	
H17W	0.0075	0.8220	0.0257	0.057*	
O10W	0.0100 (5)	0.3191 (3)	0.7221 (3)	0.0406 (8)	
H19W	−0.0044	0.3950	0.7006	0.061*	
H20W	0.1257	0.2800	0.6958	0.061*	

### Atomic displacement parameters (Å^2^)

**Table:**

	*U*^11^	*U*^22^	*U*^33^	*U*^12^	*U*^13^	*U*^23^
C1	0.0213 (19)	0.021 (2)	0.0181 (18)	−0.0063 (15)	−0.0026 (15)	0.0000 (15)
N1	0.041 (2)	0.0185 (17)	0.0163 (16)	−0.0034 (14)	−0.0012 (15)	0.0021 (13)
Ni1	0.0245 (3)	0.0148 (3)	0.0184 (3)	−0.0027 (2)	−0.0024 (2)	−0.00091 (19)
O1	0.0319 (17)	0.0426 (19)	0.0323 (16)	−0.0088 (14)	−0.0108 (13)	0.0156 (14)
C2	0.025 (2)	0.0178 (19)	0.0179 (18)	−0.0028 (15)	−0.0014 (15)	−0.0019 (14)
N2	0.0285 (18)	0.0143 (16)	0.0211 (16)	−0.0028 (13)	−0.0034 (14)	0.0013 (12)
O2	0.0295 (16)	0.0383 (18)	0.0272 (16)	−0.0074 (13)	−0.0024 (13)	0.0123 (13)
C3	0.0221 (19)	0.0179 (19)	0.0218 (19)	−0.0035 (15)	−0.0035 (15)	−0.0023 (15)
O3	0.0371 (17)	0.0234 (16)	0.0344 (17)	0.0036 (13)	0.0036 (14)	−0.0068 (12)
C4	0.028 (2)	0.021 (2)	0.0189 (19)	−0.0063 (16)	−0.0015 (16)	−0.0002 (14)
O4	0.0382 (18)	0.0188 (15)	0.0391 (17)	−0.0077 (12)	−0.0007 (14)	−0.0031 (12)
C5	0.031 (2)	0.021 (2)	0.0205 (19)	−0.0028 (16)	−0.0002 (16)	−0.0037 (15)
C6	0.023 (2)	0.017 (2)	0.024 (2)	−0.0018 (15)	−0.0034 (16)	−0.0027 (15)
C7	0.035 (2)	0.0179 (19)	0.022 (2)	−0.0049 (16)	−0.0046 (17)	0.0015 (15)
C8	0.030 (2)	0.018 (2)	0.024 (2)	−0.0036 (16)	−0.0074 (17)	−0.0021 (15)
C9	0.030 (2)	0.0166 (19)	0.0192 (19)	−0.0058 (16)	−0.0053 (16)	−0.0015 (14)
O1W	0.0327 (16)	0.0282 (16)	0.0246 (14)	−0.0124 (12)	−0.0063 (12)	0.0039 (11)
O2W	0.0297 (15)	0.0283 (16)	0.0238 (14)	−0.0103 (12)	−0.0053 (12)	0.0023 (11)
O3W	0.081 (3)	0.0219 (16)	0.0287 (17)	−0.0075 (16)	0.0088 (17)	−0.0086 (13)
O4W	0.0315 (16)	0.0221 (15)	0.0396 (17)	0.0020 (12)	−0.0139 (13)	−0.0052 (12)
O5W	0.0327 (16)	0.0227 (15)	0.0292 (15)	−0.0092 (12)	0.0025 (12)	−0.0053 (11)
O6W	0.0355 (18)	0.0310 (17)	0.0306 (16)	−0.0042 (13)	−0.0035 (13)	0.0006 (12)
O7W	0.0381 (18)	0.0401 (19)	0.0320 (17)	−0.0067 (14)	−0.0040 (14)	−0.0067 (14)
O8W	0.0349 (18)	0.039 (2)	0.052 (2)	−0.0080 (15)	−0.0099 (15)	0.0024 (15)
O9W	0.0462 (19)	0.0365 (18)	0.0310 (16)	−0.0061 (15)	−0.0090 (15)	−0.0077 (13)
O10W	0.0358 (18)	0.0334 (18)	0.051 (2)	−0.0075 (14)	−0.0088 (15)	−0.0022 (15)

### Geometric parameters (Å, °)

**Table:**

C1—C2	1.383 (5)	C5—H5	0.9300
C1—C6	1.422 (6)	C6—C8	1.506 (5)
C1—C9	1.522 (5)	C7—H7	0.9300
N1—C7	1.332 (5)	O1W—H2W	0.8400
N1—C4	1.387 (5)	O1W—H1W	0.8400
N1—H1	0.8600	O2W—H3W	0.8400
Ni1—O3W	2.029 (3)	O2W—H4W	0.8400
Ni1—O4W	2.053 (3)	O3W—H5W	0.8400
Ni1—N2	2.052 (3)	O3W—H6W	0.8400
Ni1—O2W	2.069 (3)	O4W—H7W	0.8400
Ni1—O1W	2.078 (3)	O4W—H8W	0.8400
Ni1—O5W	2.099 (3)	O5W—H9W	0.8400
O1—C9	1.242 (5)	O5W—H10W	0.8400
C2—C3	1.390 (5)	O6W—H11W	0.8400
C2—H2	0.9300	O6W—H12W	0.8400
N2—C7	1.325 (5)	O7W—H14W	0.8400
N2—C3	1.398 (5)	O7W—H13W	0.8400
O2—C9	1.247 (5)	O8W—H16W	0.8400
C3—C4	1.400 (6)	O8W—H15W	0.8400
O3—C8	1.250 (5)	O9W—H18W	0.8400
C4—C5	1.384 (6)	O9W—H17W	0.8400
O4—C8	1.263 (5)	O10W—H19W	0.8400
C5—C6	1.383 (5)	O10W—H20W	0.8400
			
C2—C1—C6	121.3 (3)	C6—C5—H5	121.1
C2—C1—C9	117.1 (3)	C4—C5—H5	121.1
C6—C1—C9	121.5 (3)	C5—C6—C1	120.4 (4)
C7—N1—C4	107.6 (3)	C5—C6—C8	118.6 (3)
C7—N1—H1	126.2	C1—C6—C8	120.9 (3)
C4—N1—H1	126.2	N2—C7—N1	113.3 (3)
O3W—Ni1—O4W	88.73 (14)	N2—C7—H7	123.4
O3W—Ni1—N2	176.19 (13)	N1—C7—H7	123.4
O4W—Ni1—N2	87.52 (13)	O3—C8—O4	124.7 (4)
O3W—Ni1—O2W	86.14 (14)	O3—C8—C6	118.0 (4)
O4W—Ni1—O2W	92.83 (12)	O4—C8—C6	117.1 (3)
N2—Ni1—O2W	94.75 (13)	O1—C9—O2	124.9 (4)
O3W—Ni1—O1W	90.63 (14)	O1—C9—C1	117.3 (3)
O4W—Ni1—O1W	176.58 (11)	O2—C9—C1	117.7 (4)
N2—Ni1—O1W	93.07 (13)	Ni1—O1W—H2W	117.9
O2W—Ni1—O1W	90.49 (11)	Ni1—O1W—H1W	106.6
O3W—Ni1—O5W	89.34 (13)	H2W—O1W—H1W	111.6
O4W—Ni1—O5W	88.84 (13)	Ni1—O2W—H3W	119.4
N2—Ni1—O5W	89.88 (13)	Ni1—O2W—H4W	115.2
O2W—Ni1—O5W	175.15 (11)	H3W—O2W—H4W	111.6
O1W—Ni1—O5W	87.79 (12)	Ni1—O3W—H5W	122.7
C1—C2—C3	118.0 (4)	Ni1—O3W—H6W	125.1
C1—C2—H2	121.0	H5W—O3W—H6W	111.9
C3—C2—H2	121.0	Ni1—O4W—H7W	113.0
C7—N2—C3	104.9 (3)	Ni1—O4W—H8W	119.4
C7—N2—Ni1	122.5 (3)	H7W—O4W—H8W	111.4
C3—N2—Ni1	132.1 (3)	Ni1—O5W—H9W	112.7
C2—C3—N2	130.8 (4)	Ni1—O5W—H10W	119.8
C2—C3—C4	120.3 (4)	H9W—O5W—H10W	111.1
N2—C3—C4	108.9 (3)	H11W—O6W—H12W	111.6
N1—C4—C5	132.6 (4)	H14W—O7W—H13W	111.7
N1—C4—C3	105.3 (3)	H16W—O8W—H15W	111.6
C5—C4—C3	122.2 (4)	H18W—O9W—H17W	111.7
C6—C5—C4	117.8 (4)	H19W—O10W—H20W	111.4

### Hydrogen-bond geometry (Å, °)

**Table:**

*D*—H···*A*	*D*—H	H···*A*	*D*···*A*	*D*—H···*A*
O10W—H20W···O1W	0.84	2.00	2.836 (4)	176
O10W—H19W···O8W^i^	0.84	1.88	2.703 (5)	166
O9W—H17W···O3^ii^	0.84	1.90	2.733 (5)	172
O9W—H18W···O10W^iii^	0.84	1.91	2.720 (5)	163
O8W—H15W···O1^iv^	0.84	1.95	2.765 (5)	163
O8W—H16W···O2	0.84	1.96	2.775 (5)	162
O7W—H13W···O8W^v^	0.84	1.93	2.754 (5)	165
O7W—H14W···O4^v^	0.84	1.91	2.734 (5)	169
O6W—H12W···O2W^vi^	0.84	2.06	2.857 (4)	159
O6W—H11W···O4^vii^	0.84	1.97	2.808 (4)	174
O5W—H10W···O4^viii^	0.84	1.96	2.800 (4)	176
O5W—H9W···O9W^iii^	0.84	1.98	2.817 (4)	173
O4W—H8W···O9W^v^	0.84	1.90	2.736 (5)	173
O4W—H7W···O3^ix^	0.84	1.94	2.709 (4)	151
O3W—H6W···O6W^viii^	0.84	1.93	2.761 (4)	172
O3W—H5W···O7W^x^	0.84	1.93	2.729 (5)	159
O2W—H4W···O1^v^	0.84	1.80	2.620 (4)	164
O2W—H3W···O10W^iv^	0.84	1.90	2.734 (5)	175
O1W—H1W···O6W^v^	0.84	1.96	2.783 (5)	168
O1W—H2W···O2^v^	0.84	1.79	2.612 (4)	166
N1—H1···O7W^xi^	0.86	1.97	2.803 (5)	162

Symmetry codes: (i) *x*−1, *y*, *z*; (ii) *x*, *y*, *z*−1; (iii) −*x*, −*y*+1, −*z*+1; (iv) *x*+1, *y*, *z*; (v) −*x*+1, −*y*+1, −*z*+1; (vi) −*x*+2, −*y*+1, −*z*+1; (vii) −*x*+1, −*y*+2, −*z*+1; (viii) *x*, *y*−1, *z*; (ix) *x*+1, *y*−1, *z*; (x) −*x*+1, −*y*, −*z*+1; (xi) *x*, *y*, *z*+1.
